# Development and validation of comprehensive nomograms from the SEER database for predicting early mortality in metastatic rectal cancer patients

**DOI:** 10.1186/s12876-024-03178-y

**Published:** 2024-02-26

**Authors:** Yanli Li, Ting Tao, Yun Liu

**Affiliations:** Department of Pharmacy, The First People’s Hospital of Lianyungang, Affiliated Hospital of Xuzhou Medical University, 222061 Lianyungang, China

**Keywords:** Nomogram, Early mortality, Risk factors, SEER, Metastatic rectal cancer

## Abstract

**Background:**

Metastatic rectal cancer is an incurable malignancy, which is prone to early mortality. We aimed to establish nomograms for predicting the risk of early mortality in patients with metastatic rectal cancer.

**Methods:**

In this study, clinical data were obtained from the Surveillance, Epidemiology, and End Results (SEER) database.We utilized X-tile software to determine the optimal cut-off points of age and tumor size in diagnosis. Significant independent risk factors for all-cause and cancer-specific early mortality were determined by the univariate and multivariate logistic regression analyses, then we construct two practical nomograms. In order to assess the predictive performance of nomograms, we performed calibration plots, time-dependent receiver-operating characteristic curve (ROC), decision curve analysis (DCA) and clinical impact curve (CIC).

**Results:**

A total of 2570 metastatic rectal cancer patients were included in the study. Multivariate logistic regression analyses revealed that age at diagnosis, CEA level, tumor size, surgical intervention, chemotherapy, radiotherapy, and metastases to bone, brain, liver, and lung were independently associated with early mortality of metastatic rectal cancer patients in the training cohort. The area under the curve (AUC) values of nomograms for all-cause and cancer-specific early mortality were all higher than 0.700. Calibration curves indicated that the nomograms accurately predicted early mortality and exhibited excellent discrimination. DCA and CIC showed moderately positive net benefits.

**Conclusions:**

This study successfully generated applicable nomograms that predicted the high-risk early mortality of metastatic rectal cancer patients, which can assist clinicians in tailoring more effective treatment regimens.

## Introduction

Colorectal cancer remains one of the predominant malignancies within the digestive system. Alarmingly, its incidence is on the rise in individuals younger than 50 years [1]. In the Western world, rectal cancer constitutes approximately 25% of all large bowel cancers. Projections suggest that by 2030, the incidence of rectal cancer in the 20–34 ages will surge by 124% [2]. Treatment modalities for rectal cancer are dictated by the clinical tumor stage and are continually refined based on the latest oncological guidelines [3]. For early-stage rectal cancer, a standardized surgical approach focusing on tumor resection is the cornerstone. However, for patients diagnosed with locally advanced rectal cancer, neoadjuvant chemoradiotherapy (NCRT) is typically prescribed before surgical intervention [4]. While NCRT has proven efficacious in augmenting the likelihood of curative resection and promoting tumor regression, it has not demonstrated a discernible impact on overall survival [5]. The principal threat to survival stems from distant metastases (DM), which can manifest in vital organs such as the liver, lungs, brain, and bones [6]. Strikingly, metastatic tumors are identified in nearly half of all rectal cancer patients, marking a significant contributor to the grim prognosis associated with this disease and its constrained 5-year survival rate [7]. Hence, accurate early prediction of mortality is paramount. Such insights can empower clinicians to swiftly identify high-risk patients, ultimately enhancing the prognosis for those with metastatic rectal cancer.

The American Joint Committee on Cancer (AJCC) staging system is widely adopted in contemporary clinical practice for determining treatment strategies and prognostic evaluation of rectal cancer patients. However, discrepancies have been observed in clinical outcomes among patients with identical staging, even when subjected to similar treatment regimens. A plausible explanation for this variation is the AJCC system’s primary focus on tumor characteristics, such as stage, while largely overlooking vital clinicopathological features. These overlooked features encompass demographic attributes, histological variants, and specific clinical treatments, all of which can significantly influence patient outcomes.

Currently, nomograms have emerged as a novel tool for predicting prognosis and guiding treatment decisions in various types of cancer, as demonstrated in previous studies [8]. The performance of nomograms has shown promise compared to the traditional TNM staging system. However, it is important to note that while many nomogram models have been developed to predict prognostic information and overall survival in patients with rectal cancer, there is a noticeable gap regarding well-established nomograms specifically designed for predicting early mortality in patients with metastatic rectal cancer. Furthermore, existing studies in this regard have often relied on small sample sizes or regionally limited patient cohorts, which hinders their broader clinical applicability [9, 10]. To the best of our knowledge, there have been no reports of nomograms based on risk models for predicting the incidence of early mortality in metastatic rectal cancer patients. Therefore, the development of a nomogram for predicting premature mortality has become increasingly important, particularly for patients with DM. Such a tool can significantly aid clinicians in tailoring more effective treatment regimens for these individuals.

The Surveillance, Epidemiology, and End Results (SEER) database, maintained by the National Cancer Institute, serves as a valuable resource for studying the epidemiological characteristics of cancer. This database encompasses approximately 28% of the United States population and provides extensive clinicopathological information along with follow-up data for cancer patients. Therefore, the primary objective of our study is to construct user-friendly and comprehensive nomograms that can predict the likelihood of early mortality among metastatic rectal cancer patients. To achieve this, we utilized data gathered from the SEER database, allowing us to examine the various factors associated with early mortality following the initial diagnosis in this patient population.

## Materials and methods

### Population

**Fig. 1:**
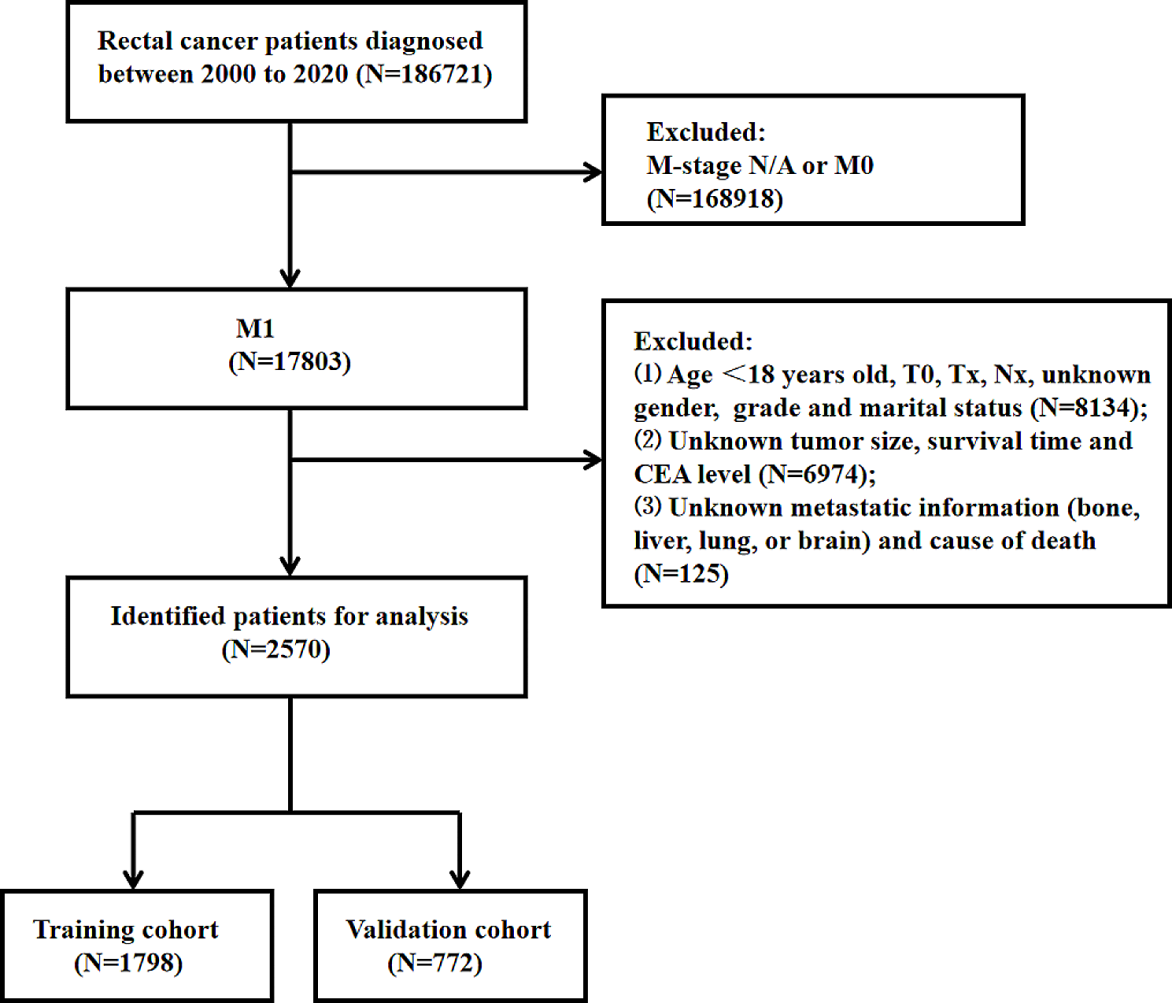
Flowchart for selection of metastatic rectal cancer patients

The SEER database, supported by the National Cancer Institute, is currently one of the most valuable cancer registry databases worldwide. To extract clinical information, SEER*Stat software (Version 8.4.2; https://seer.cancer.gov/seerstat/) was utilized. Given the public nature of the SEER database’s data, our study did not necessitate obtaining informed consent from patients. For our research, the 3rd Edition of the International Classification of Diseases for Oncology (ICD-O-3) criteria was applied to identify cases related to rectal cancer (codes C19.9 and C20.9). Our study included 186,721 cases from the SEER Research data, encompassing 17 registries and data from November 2022 (covering the period from 2000 to 2020). Our focus was on patients with primary tumors localized in the rectum. The schematic representation detailing the inclusion and exclusion criteria for metastatic rectal cancer patients can be viewed in Fig. 1.

From the SEER database, 19 clinical variables were extracted: age at diagnosis, gender, race, grade, histological classification, T stage (AJCC 7th version), N stage (AJCC 7th version), carcinoembryonic antigen (CEA) level, tumor size, cause of death, survival duration, surgery received (including primary and/or distant metastases sites), chemotherapy, radiotherapy, marital status, and specific sites of DM (including bone, brain, liver, and lung). The inclusion criteria included: (1) the M stage of primary tumor was M1, which was identified as simultaneous metastases of rectal cancer. (2) Patient age was 18 years or older. (3) Rectal cancer was the only primary carcinoma. (4) Complete clinicopathological records were available with a minimum follow-up period of one month. The exclusion criteria were: (1) Absence of important clinical data, such as gender, race, grade, histological type, TNM stages and tumor size. (2) Incomplete information on specific metastatic sites (bone, liver, lung, or brain). (3) Indeterminate survival duration or survival status. Additionally, the term “tumor size” in this study specifically denotes the primary tumor size. An “all-cause early mortality” in patients with metastatic rectal cancer was characterized as any mortality occurring within the initial 3 months post-diagnosis, and a “cancer-specific early mortality” was specified as an early mortality attributed to cancer-related causes within the same 3-month window since the of diagnosis rectal cancer. The Sample Function of R software (version 4.3.1) was employed to design and validate the nomograms. This allowed us to randomly partition the cohort of metastatic rectal cancer patients into training and validation groups at a 7:3 ratio.

### Nomogram construction and statistical analysis

The categorical variables within the study population are presented as numbers and percentages (N, %). For determining optimal cut-off points for age at diagnosis and tumor size, we employed the X-tile software (version 3.6.1). The age brackets were defined as ≤ 57, 58–75, and ≥ 76 years, while tumor size categories were ≤ 55 mm, 56–85 mm, and ≥ 86 mm. Univariate and multivariate logistic regression analyses were employed to identify independent risk factors contributing to early mortality in metastatic rectal cancer patients. Subsequently, predictive nomogram models, informed by the identified risk factors, were formulated to anticipate both all-cause and cancer-specific early mortalitys in this patient cohort.

### Performance and validation of nomograms

The efficacy of the nomograms was assessed in both the training and validation cohorts using several metrics. The concordance index (C-index) was utilized to determine the discrimination of the nomogram model. To ensure the nomograms’ precision and reliability, calibration curves, reinforced by 1000 bootstrap resamples, were plotted. The predictive power of the nomograms was further appraised using the receiver operating characteristic (ROC) curve, with emphasis on the area under the ROC curve (AUC) values. Furthermore, decision curve analysis (DCA) and clinical impact curve (CIC) were conducted to quantify the clinical relevance and usefulness of the nomograms. All statistical analyses were executed in the R software environment (version 4.3.1) via R Studio. A two-sided P-value of less than 0.05 was deemed statistically significant.

## Results

### Patient characteristics

From our selected criteria, we identified 2,570 metastatic rectal cancer patients for inclusion in this study. These participants were randomly allocated into a training cohort (*n* = 1,798) and a validation cohort (*n* = 772). Of the total, 1,564 patients (60.86%) were male, while 1,006 (39.14%) were female. The average age was 58.56 ± 12.84 years. The majority of the patients were Caucasian, accounting for 79.34% (*n* = 2,039), and over half of the patients were under 60 years old, comprising 56.85% (*n* = 1,461). In terms of treatment modalities, surgery was undergone by 65.10% (*n* = 1,673) of the patients, chemotherapy was administered to 85.37% (*n* = 2,194), and radiotherapy was opted for by 39.81% (*n* = 1,023). When we evaluated the metastatic sites, we found that 4.86% of patients had bone metastases, 0.89% had brain metastases, 72.1% presented with liver metastases, and 26.61% had lung metastases. In this study, the observed incidences of cancer-specific early mortality and all-cause early mortality were 6.96% (*N* = 179) and 7.82% (*N* = 201), respectively. Notably, patients older than 76 years displayed a markedly higher incidence of early mortality from all causes at 20.8%, significantly surpassing other age groups (*p* < 0.05). Additionally, when examining tumor grade, there were discernible differences in early mortality rates. Grade I tumors had the lowest incidence at 5.19%, followed by Grade II at 7.11% and Grade III at 10.26%. The highest early mortality incidence was observed in patients with Grade IV metastatic rectal cancer, standing at 10.8%. Pertinently, liver metastasis was predominant, with 78.11% of early mortality linked to this site, making it the most frequent metastatic association with early mortality. Notably, the demographic and clinical attributes were evenly distributed between the training and validation cohorts, ensuring no significant variances. The distribution of patient characteristics for both cohorts is detailed in Table 1.

**Table 1:** Demographics and clinicopathological characteristics of 2570 patients

		Training cohort (*N* = 1798)	Validation cohort (*N* = 772)	*p*
Age (years)				0.100
	≤ 57	889(49.44%)	347(44.95%)	
	58 ~ 75	719(39.99%)	341(44.17%)	
	≥ 76	190(10.57%)	84(10.88%)	
Race				0.843
	White	1421(79.03%)	618(80.05%)	
	Black	169(9.40%)	69(8.94%)	
	Other	208(11.57%)	85(11.01%)	
Gender				0.978
	Male	1095(60.90%)	469(60.75%)	
	Female	703(39.10%)	303(39.25%)	
Histological type				0.182
	Not adenocarcinoma	286(15.91%)	140(18.13%)	
	Adenocarcinoma	1512(84.09%)	632(81.87%)	
Grade				0.422
	Well differentiated: I	89(4.95%)	46(5.96%)	
	Moderately differentiated: II	1315(73.14%)	542(70.21%)	
	Poorly differentiated: III	339(18.85%)	161(20.85%)	
	Undifferentiated;anaplastic:IV	55(3.06%)	23(2.98%)	
T Stage				0.725
	T1	193(10.73%)	91(11.79%)	
	T2	79(4.39%)	29(3.76%)	
	T3	1067(59.34%)	449(58.15%)	
	T4	459(25.54%)	203(26.30%)	
N Stage				0.822
	N0	463(25.75%)	204(26.42%)	
	N1	768(42.71%)	334(43.26%)	
	N2	567(31.54%)	234(30.32%)	
CEA level				0.462
	Negative	403(22.41%)	184(23.83%)	
	Positive	1395(77.59%)	588(76.17%)	
Tumor size				0.912
	≤ 55	1054(58.62%)	454(58.81%)	
	56 ~ 85	522(29.03%)	219(28.37%)	
	≥ 86	222(12.35%)	99(12.82%)	
Surgery				0.853
	No	625(34.76%)	272(35.23%)	
	Yes	1173(65.24%)	500(64.77%)	
Chemotherapy				0.665
	No	259(14.4%)	117(15.16%)	
	Yes	1539(85.6%)	655(84.84%)	
Radiotherapy				1
	No	1082(60.18%)	465(60.23%)	
	Yes	716(39.82%)	307(39.77%)	
Marital status				0.613
	Unmarried	799(44.44%)	334(43.26%)	
	Married	999(55.56%)	438(56.74%)	
Bone metastases				0.429
	No	1715(95.38%)	730(94.56%)	
	Yes	83(4.62%)	42(5.44%)	
Brain metastases				0.101
	No	1786(99.33%)	761(98.58%)	
	Yes	12(0.67%)	11(1.42%)	
Liver metastases				0.343
	No	512(28.48%)	205(26.55%)	
	Yes	1286(71.52%)	567(73.45%)	
Lung metastases				0.438
	No	1311(72.91%)	575(74.48%)	
	Yes	487(27.09%)	197(25.52%)	

### Risk factors for early mortality in the training cohort

Univariate logistic regression analysis revealed that variables such as age at diagnosis, T stage, CEA level, tumor size, surgery, chemotherapy, radiotherapy, and metastases to the bone, liver, and brain were all significantly correlated with both all-cause and cancer-specific early mortality(Table 2). Interestingly, marital status was exclusively linked with all-cause early mortality (Table 3). Upon further evaluation using multivariable logistic regression, factors such as advanced age at diagnosis, increased tumor size, undergoing chemotherapy and radiotherapy, along with metastases to the bone, liver, and brain were all positively correlated with both forms of early mortality. Meanwhile, CEA levels and surgery emerged as independent risk factors uniquely for all-cause early mortality.

**Table 2:** Univariate and multivariate analysis of all-cause early mortality in the training cohort

Variables	Univariate analysis				Multivariate analysis		
	OR	95% Cl	*P*		OR(95%)	95%Cl	*P*
Age (years)							
≤ 57	Ref				Ref		
58 ~ 75	2.173	1.436–3.333	<0.001		1.319	0.827–2.115	0.213
≥ 76	6.535	4.067–10.549	<0.001		2.958	1.683–5.192	<0.001
Race							
White	Ref						
Black	1.333	0.754–2.225	0.295				
Other	0.86	0.464–1.481	0.608				
Gender							
Female	Ref						
Male	1.004	0.708–1.435	0.981				
Histological type							
Adenocarcinoma	Ref						
Not adenocarcinoma	1.153	0.719–1.782	0.538				
Grade							
Well differentiated: I	Ref						
Moderately differentiated: II	1.004	0.462–2.636	0.992				
Poorly differentiated: III	1.851	0.814–4.993	0.176				
Undifferentiated;anaplastic:IV	0.384	1.694–0.504	0.384				
T Stage							
T1	Ref				Ref		
T2	0.499	0.163–1.267	0.176		0.532	0.151–1.601	0.289
T3	0.511	0.315–0.858	0.008		0.82	0.442–1.557	0.536
T4	0.784	0.463–1.357	0.372		0.98	0.502–1.945	0.954
N Stage							
N0	Ref						
N1	0.861	0.556–1.348	0.508				
N2	1.223	0.789–1.916	0.372				
CEA level							
Positive	Ref				Ref		
Negative	0.359	0.196–0.61	<0.001		0.503	0.258–0.917	0.0326
Tumor size							
≤ 55	Ref				Ref		
56 ~ 85	1.593	1.073–2.351	0.02		1.205	0.767–1.882	0.414
≥ 86	2.458	1.532–3.867	<0.001		2.556	1.46–4.421	<0.001
Surgery							
Yes	Ref				Ref		
No	2.149	1.521–3.041	<0.001		1.758	1.112–2.775	0.015
Chemotherapy							
Yes	Ref				Ref		
No	13.91	9.6–20.328	<0.001		10.112	6.616–15.613	<0.001
Radiotherapy							
Yes	Ref				Ref		
No	3.005	1.984–4.712	<0.001		1.772	1.083–2.973	0.026
Marital status							
Married	Ref				Ref		
Unmarried	1.51	1.07–2.137	0.019		1.078	0.721–1.61	0.715
Bone metastases							
Yes	Ref				Ref		
No	0.329	0.19–0.603	<0.001		0.319	0.165–0.644	<0.001
Brain metastases							
Yes	Ref						
No	0.936	0.18–17.165	0.949				
Liver metastases							
Yes	Ref				Ref		
No	0.629	0.406–0.946	0.032		0.536	0.323–0.867	0.013
Lung metastases							
Yes	Ref				Ref		
No	0.44	0.31–0.626	<0.001		0.488	0.320–0.745	<0.001

**Table 3:** Univariate and multivariate analysis of cancer-specific early mortality in the training cohort

Variables	Univariate analysis				Multivariate analysis		
	OR	95%Cl	*P*		OR	95%Cl	*P*
Age (years)							
≤ 57	Ref				Ref		
58 ~ 75	1.824	1.174–2.861	0.008		1.092	0.668–1.792	0.727
≥ 76	6.302	3.87–10.305	<0.001		2.956	1.664–5.243	<0.001
Race							
White	Ref						
Black	1.219	0.653–2.119	0.506				
Other	0.827	0.424–1.475	0.546				
Gender							
Female	Ref						
Male	1.018	0.702–1.488	0.927				
Histological type							
Adenocarcinoma	Ref						
Not adenocarcinoma	1.367	0.848–2.128	0.181				
Grade							
Well differentiated: I	Ref						
Moderately differentiated: II	1.045	0.453–3.034	0.926				
Poorly differentiated: III	2.058	0.855–6.126	0.142				
Undifferentiated;anaplastic:IV	1.68	0.447–6.317	0.43				
T Stage							
T1	Ref				Ref		
T2	0.461	0.131–1.269	0.171		0.458	0.113–1.487	0.225
T3	0.552	0.331–0.957	0.027		0.857	0.451–1.674	0.643
T4	0.736	0.418–1.329	0.296		0.885	0.437–1.822	0.737
N Stage							
N0	Ref						
N1	0.799	0.499–1.29	0.352				
N2	1.274	0.805–2.042	0.306				
CEA level							
Positive	Ref				Ref		
Negative	0.42	0.228–0.717	0.003		0.621	0.317–1.139	0.142
Tumor size							
≤ 55	Ref				Ref		
56 ~ 85	1.559	1.025–2.355	0.036		1.213	0.756–1.932	0.418
≥ 86	2.364	1.429–3.82	<0.001		2.578	1.432–4.57	0.001
Surgery							
Yes	Ref				Ref		
No	1.973	1.367–2.849	<0.001		1.503	0.929– 2.422	0.095
Chemotherapy							
Yes	Ref				Ref		
No	12.395	8.411–18.423	<0.001		8.812	5.668– 13.834	<0.001
Radiotherapy							
Yes	Ref				Ref		
No	3.283	2.091–5.387	<0.001		2.012	1.1913–3.516	0.011
Marital status							
Married	Ref						
Unmarried	1.271	0.882–1.833	0.197				
Bone metastases							
Yes	Ref				Ref		
No	0.308	0.175–0.575	<0.001		0.285	0.145–0.584	<0.001
Brain metastases							
Yes	Ref						
No	430,000	3.963e-08–NA	0.975				
Liver metastases							
Yes	Ref				Ref		
No	0.583	0.361–0.906	0.021		0.486	0.281–0.81	0.007
Lung metastases							
Yes	Ref				Ref		
No	0.406	0.281–0.589	<0.001		0.427	0.275–0.662	<0.001

### Nomograms construction

**Fig. 2:**
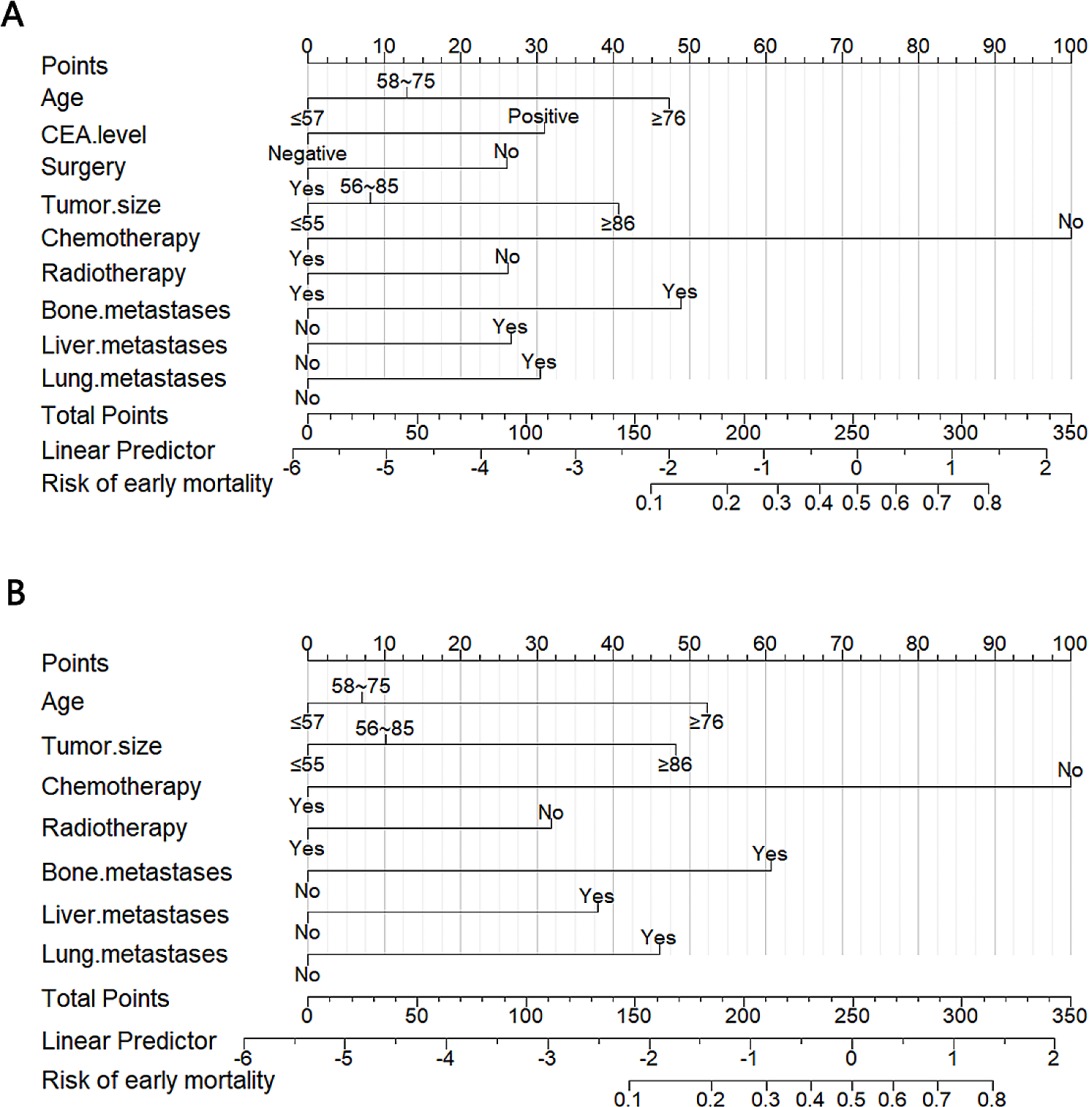
The nomograms of early mortality in patients with metastatic rectal cancer patients. (**A**) The all-cause early mortality; (**B**) The cancer-specific early mortality

Based on the results of the univariate and multivariate logistic regression analyses concerning early mortality in patients with metastatic rectal cancer, we incorporated all significant variables, including age at diagnosis, CEA level, tumor size, surgery, chemotherapy, radiotherapy, and metastases to the bone, liver, and lung. Using these parameters, the predictive nomograms were constructed for both all-cause and cancer-specific early mortality (Fig. 2A-B). Each risk factor in the nomograms is assigned a score. By counting the scores for each variable, one can determine the overall probability of early mortality in patients with metastatic rectal cancer.

### Evaluation and validation of the nomograms

**Fig. 3:**
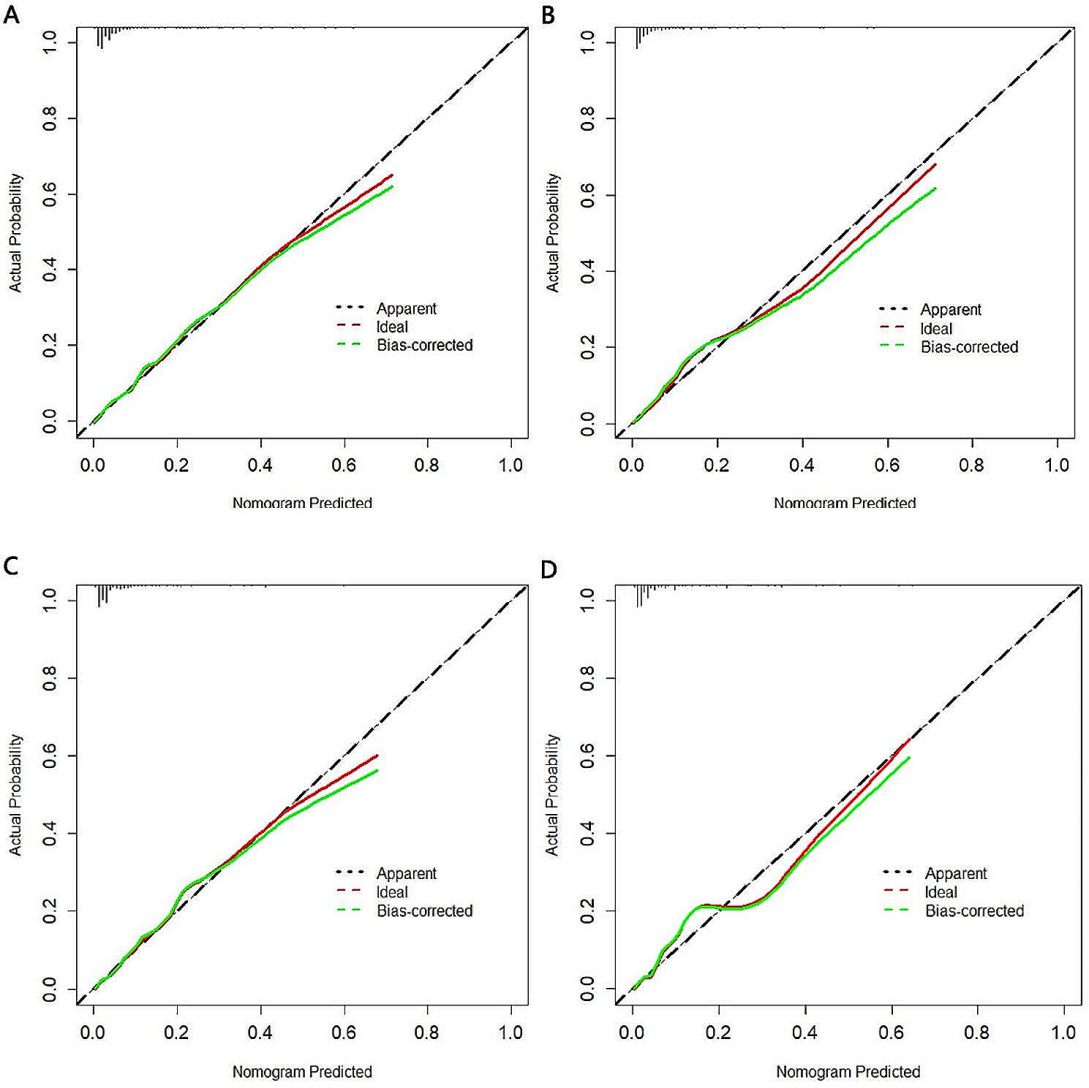
The Calibration curves by bootstrapping with 1,000 resamples for the nomograms. (**A**) The training cohort of all-cause early mortality; (**B**) The validation cohort of all-cause early mortality; (**C**) The training cohort of cancer-specific early mortality; (**D**) The validation cohort of cancer-specific early mortality

**Fig. 4:**
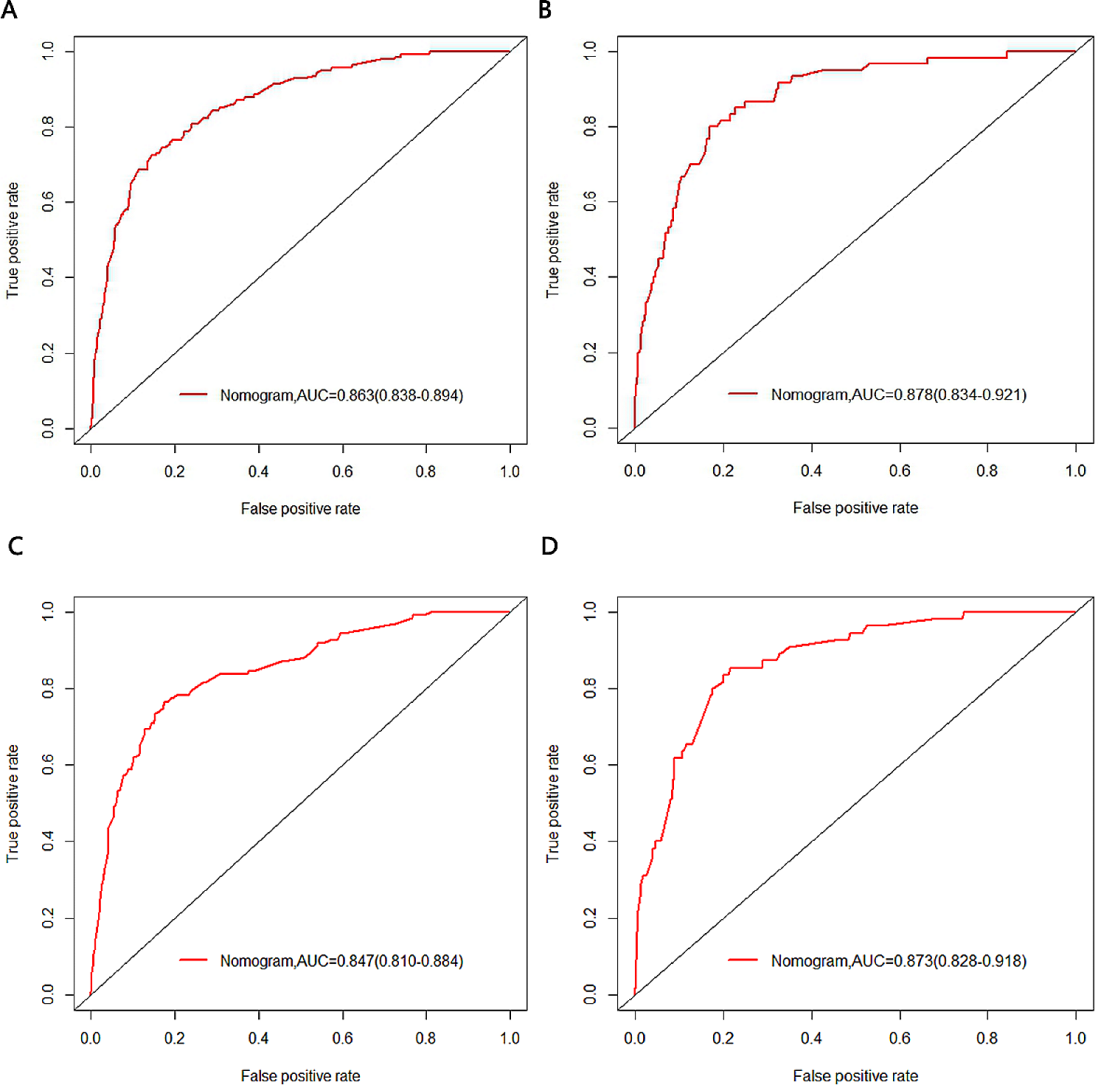
The receiver operating characteristic (ROC) curves for the nomograms. (**A**) The training cohort of all-cause early mortality; (**B**) The validation cohort of all-cause early mortality; (**C**) The training cohort of cancer-specific early mortality; (**D**) The validation cohort of cancer-specific early mortality

**Fig. 5:**
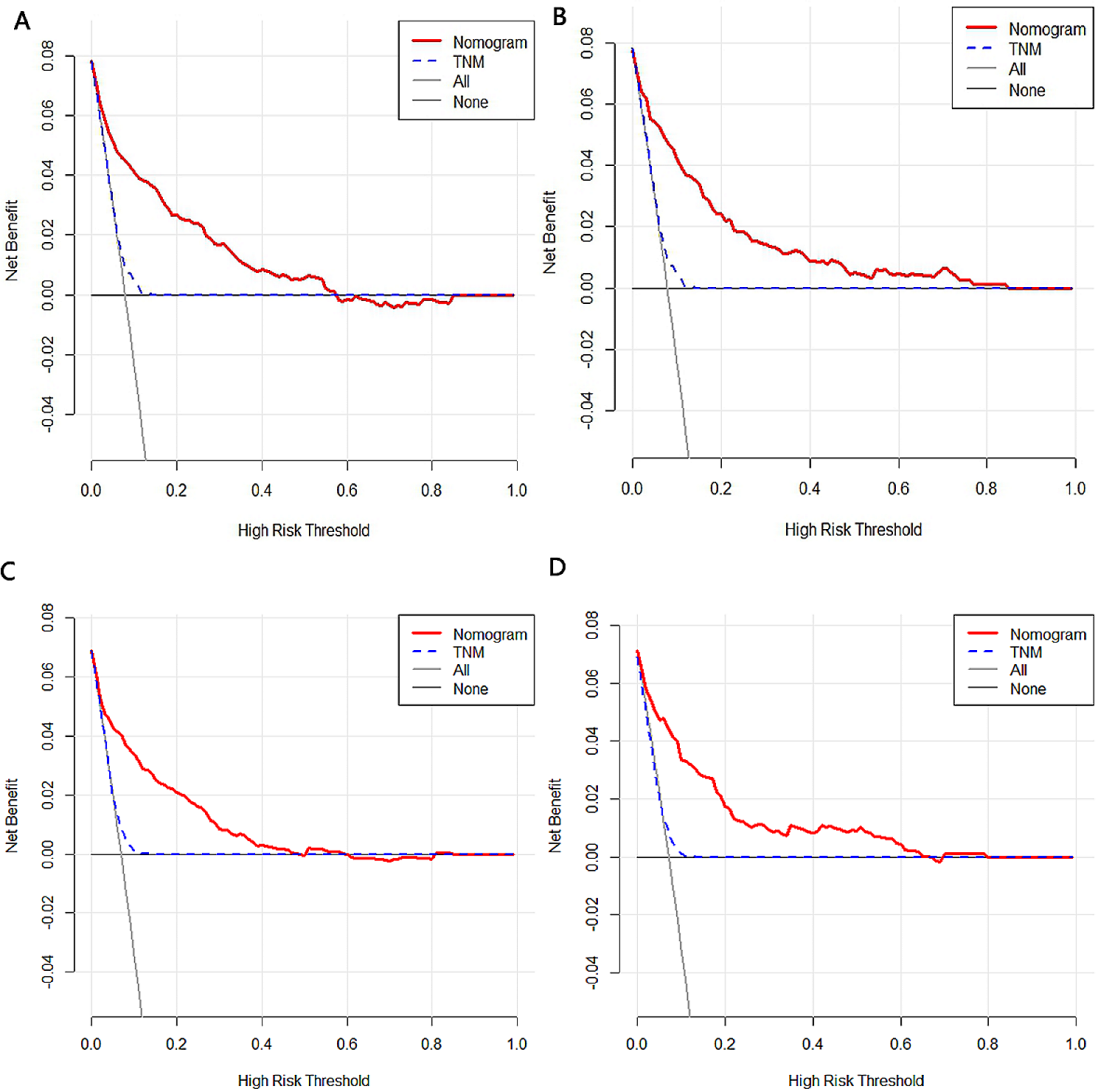
The decision curve analysis (DCA) curves for the nomograms. (**A**) The training cohort of all-cause early mortality; (**B**) The validation cohort of all-cause early mortality; (**C**) The training cohort of cancer-specific early mortality; (**D**) The validation cohort of cancer-specific early mortality

**Fig. 6:**
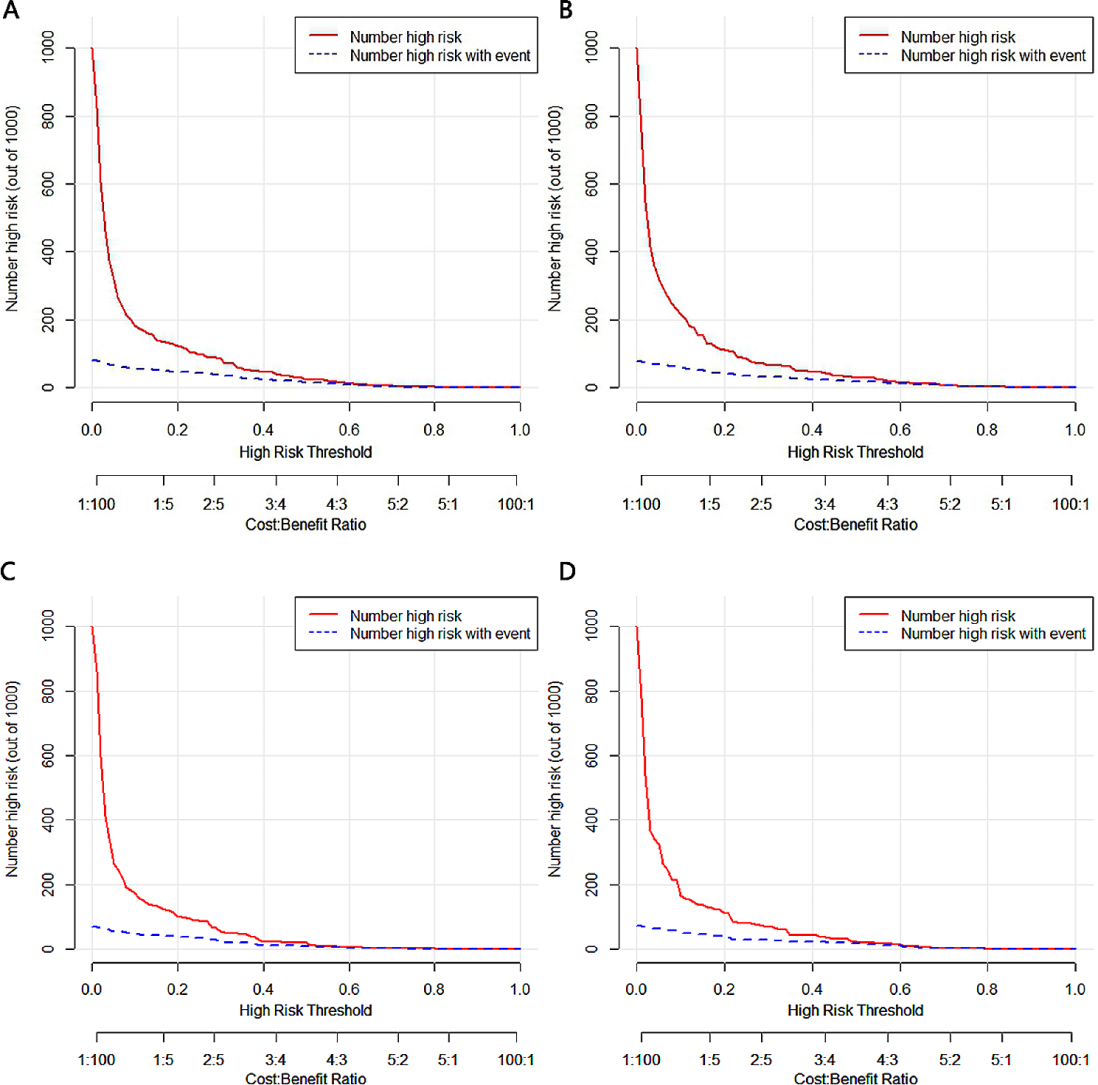
The clinical impact curve (CIC) curves for nomograms. (**A**) The training cohort of all-cause early mortality; (**B**) The validation cohort of all-cause early mortality; (**C**) The training cohort of cancer-specific early mortality; (**D**) The validation cohort of cancer-specific early mortality

In the training cohort, the nomograms demonstrated robust predictive accuracy for both all-cause and cancer-specific early mortality. The calibration curves revealed that the prediction curves closely aligned with the diagonal line, denoting accurate prediction for both early mortality categories in the training and validation cohorts(Fig. 3). For all-cause early mortality, the AUC was 0.863 (Figs. 4A and 95% CI: 0.838–0.894) in the training group. Similarly, the AUC for cancer-specific early mortality was 0.847 (Figs. 4C and 95% CI: 0.810–0.884) in the training group, indicating strong discrimination. These findings suggested that the constructed nomograms possessed outstanding discriminative ability in forecasting early mortality in patients with metastatic rectal cancer. Upon validation, the AUC values for predicting all-cause and cancer-specific early mortality were 0.878 (95% CI, 0.834–0.921, Fig. 4B) and 0.873 (95% CI, 0.828–0.918, Fig. 4D) respectively. Furthermore, the DCA and CIC for the prediction nomograms are illustrated in Figs. 5 and 6. When contrasted with the TNM staging system, both DCA and CIC curves for the training and validation cohorts revealed that the predictive nomograms offered greater net benefits and favorable clinical outcomes. These findings underscored their superior clinical applicability for predicting early mortality in patients with metastatic rectal cancer.

## Discussion

Rectal cancer ranks as the eighth most prevalent cancer worldwide and stands ninth in terms of cancer-related mortality [11]. While advancements like neoadjuvant therapy, precision surgery, and immunotherapy have led to a decline in its incidence and mortality, the heterogeneous nature of rectal cancer means that many patients, particularly those presenting with distant metastasis at diagnosis, still struggle to receive an accurate prognosis [12].

In recent years, there has been a growing interest in understanding the median survival time and prognostic factors for rectal cancer patients [13, 14]. However, much of this research has centered on the 1-, 3-, and 5-year overall survival rates for rectal cancer. Surprisingly, limited attention has been given to the early mortality or its associated risk factors among patients with metastatic rectal cancer. Given this gap, there’s a pressing need for predictive models to pinpoint those at heightened risk of early mortality, aiming for improved patient outcomes.

Creating nomograms to predict the risk of both all-cause and cancer-specific early mortality in metastatic rectal cancer patients holds significant clinical importance. In this study, utilizing a combination of clinicopathological features, treatment data, and metastatic information, we constructed predictive nomograms. These were designed to outline the incidence and factors associated with early mortality in patients with metastatic rectal cancer. Prior research has highlighted certain characteristics, such as age, surgical intervention, chemotherapy, and distant metastasis, as being statistically linked to the prognosis of colorectal cancer patients [15].

This study was the first to confirm that these identified risk factors also pertained to the early mortality of metastatic rectal cancer patients. Through multivariate analyses, we discerned nine critical independent risk determinants: age at diagnosis, CEA level, tumor size, surgical intervention, chemotherapy, radiotherapy, and metastases to bone, brain, liver, and lung. Notably, factors such as advanced age, larger tumor size, and the presence of distant metastasis were linked with an elevated odds ratio for both all-cause and cancer-specific early mortality. Conversely, a lower CEA level, surgical interventions, chemotherapy, and radiotherapy correlated with a reduced odds ratio for all-cause early mortality. Moreover, both chemotherapy and radiotherapy emerged as pertinent factors influencing cancer-specific early mortality.

Previous studies have established that age is a pivotal prognostic factor for colorectal cancer patients, with advancing age often correlating with poorer outcomes [16]. Han et al. [9] have demonstrated that patients aged 65 and above generally face a bleaker prognosis and significant decline in quality of life when dealing with metastatic colorectal cancer. While advanced and highly invasive tumors are recognized contributors to early mortality, few studies have pinpointed specific risk factors for early mortality in rectal cancer patients [17]. Our research determined that being older than 75 years served as an independent risk factor for early mortality, aligning with previous observations.

Besides age, the prognosis for colorectal cancer patients has been extensively linked to factors such as tumor grade, stage, and histological type. Many researchers have developed nomograms based on these parameters [18, 19]. Yet, in our current study, we diverged from this widely accepted perspective. For the first time, we identified that CEA level, tumor size, surgery, chemotherapy, and radiotherapy were risk factors for early mortality in patients with metastatic rectal cancer.

The primary treatment approach for metastatic rectal cancer has traditionally centered around chemotherapy. Neoadjuvant chemoradiotherapy followed by adjuvant chemotherapy has been recommended to prolong the survival of metastatic rectal cancer patients [7]. These recommendations are typically based on tumor characteristics, biomarker assessments, and regional variations in treatment preferences. In our research, both chemotherapy and radiotherapy emerged as significant factors influencing early mortality, echoing many findings in existing literature. Yet, the role of surgery as a predictor in our predictive model presented an interesting variance. While certain studies have reported that patients with advanced rectal cancer, when subjected to surgical resection following neoadjuvant treatments, experience a significant drop in local recurrence rates and an enhancement in survival outcomes [20], our study found the role of surgery in treating metastatic rectal cancer to be a matter of debate. In our findings, surgery wasn’t a critical predictor for cancer-specific early mortality in metastatic rectal cancer patients. However, the absence of surgical intervention was linked with a 1.758-fold increase in the risk of all-cause early mortality. This suggested that surgical interventions necessitated a more individualized consideration for each patient.

While individual predictive factors can sometimes be discriminatory, their integration can offer a more nuanced understanding. Song et al. [21] highlighted that the CEA level was a significant prognostic factor, closely associated with overall survival in patients with resected locally advanced rectal cancer who underwent neoadjuvant chemoradiotherapy. In our study, although the CEA level was linked to all-cause early mortality, it wasn’t correlated with cancer-specific early mortality in patients with metastatic rectal cancer. This observation underscores the importance of including the CEA level in the predictive nomogram, aiding in accurately identifying patients with a heightened risk of all-cause early mortality. Several studies reported the a good prognosis with a wide range of overall survival outcomes in rectal cancer patients with lung metastases [22, 23], which was not confirmed in the present study. We confirmed that lung metastases was the risk factor of early mortality for metastatic rectal cancer patients. It’s because most of stage IV rectal cancer patients in our study were with multiple metastatic sites. Approximately 59.06% of lung metastases rectal cancer patients have synchronic live metastatic disease, 6.87% were bone metastases, 1.61% were brain metastases.

From the gathered data, we constructed individualized prediction nomograms for early mortality, and subsequently assessed their predictive performance. Our results showcased that the nomograms possessed a commendable C-index for predicting both all-cause and cancer-specific early mortality, recorded at 0.863 and 0.847, respectively, highlighting a strong predictive capability. Additionally, DCA curves revealed that, across a broad spectrum of threshold probabilities, our nomograms offered superior net benefits compared to the conventional TNM staging system. These observations were further corroborated in the validation set, underscoring the reliability and robustness of our nomograms. The predictive nomograms devised in this study offer clinicians a robust tool, enhancing clinical decision-making and tailoring interventions to improve outcomes for metastatic rectal cancer patients based on their specific early mortality risk factors.

However, our study is not without limitations. Firstly, both the training and validation sets were sourced from the SEER database, which is retrospective in nature. Consequently, the inherent biases of retrospective analyses cannot be overlooked. Secondly, the SEER database did not encompass all potentially influential clinical details that could impact early mortality outcomes in metastatic rectal cancer patients. For instance, data pertaining to surgical margins, the specific adjuvant chemoradiotherapy regimen used, the precise surgical resection method, socio-economic determinants, cultural beliefs, and key genetic markers like KRAS and BRAF mutations are absent from the SEER dataset. Lastly, given that both our training and validation cohorts are retrospective, the true reliability and applicability of our predictive nomograms would benefit from validation in a prospective cohort. Despite these caveats, our predictive models demonstrate commendable efficacy in forecasting early mortality in metastatic rectal cancer patients, thereby assisting clinicians in formulating individualized therapeutic approaches.

## Conclusion

In conclusion, our study identified several key independent risk factors for early mortality in metastatic rectal cancer patients, namely age at diagnosis, CEA level, tumor size, surgical intervention, chemotherapy, radiotherapy, and metastases to the bone, brain, liver, and lungs. Crucially, based on these determinants, we developed well-calibrated nomograms to estimate the likelihood of both all-cause and cancer-specific early mortality. Demonstrating substantial accuracy and dependability, these nomograms can greatly assist clinicians in formulating personalized treatment strategies, ultimately enhancing survival outcomes for patients with metastatic rectal cancer.

## Data Availability

The data that support the findings of this study are available from the Surveillance, Epidemiology, and End Results (SEER) database at http://www.seer.cancer.gov.
